# *In silico* prediction of monovalent and chimeric tetravalent vaccines for prevention and treatment of dengue fever

**DOI:** 10.7555/JBR.31.20160109

**Published:** 2017-12-02

**Authors:** Vijayakumar Subramaniyan, Ramesh Venkatachalam, Prabhu Srinivasan, Manogar Palani

**Affiliations:** Computational Phytochemistry Laboratory P.G. and Research Department of Botany and Microbiology, A.V.V.M. Sri Pushpam College (Autonomous), Poondi, Thanjavur district, Tamil Nadu 613503, India.; Computational Phytochemistry Laboratory P.G. and Research Department of Botany and Microbiology, A.V.V.M. Sri Pushpam College (Autonomous), Poondi, Thanjavur district, Tamil Nadu 613503, India.; Computational Phytochemistry Laboratory P.G. and Research Department of Botany and Microbiology, A.V.V.M. Sri Pushpam College (Autonomous), Poondi, Thanjavur district, Tamil Nadu 613503, India.; Computational Phytochemistry Laboratory P.G. and Research Department of Botany and Microbiology, A.V.V.M. Sri Pushpam College (Autonomous), Poondi, Thanjavur district, Tamil Nadu 613503, India.

**Keywords:** dengue serotypes, dengue virus, vaccine, *E*-proteins, MHC I and II

## Abstract

Reverse vaccinology method was used to predict the monovalent peptide vaccine candidate to produce antibodies for therapeutic purpose and to predict tetravalent vaccine candidate to act as a common vaccine to cover all the dengue virus serotypes. Envelope (*E*)-proteins of DENV-1-4 serotypes were used for vaccine prediction using NCBI, Uniprot/Swissprot, Swiss-prot viewer, VaxiJen V2.0, TMHMM, BCPREDS, Propred-1, Propred and MHC Pred. *E*-proteins of DENV-1-4 serotypes were identified as antigen from which T cell epitopes, through B cell epitopes, were predicted to act as peptide vaccine candidates. Each selected T cell epitope of *E*-protein was confirmed to act as vaccine and to induce complementary antibody against particular serotype of dengue virus. Chimeric tetravalent vaccine was formed by the conjugation of four vaccines, each from four dengue serotypes to act as a common vaccine candidate for all the four dengue serotypes. It can be justifiably concluded that the monovalent 9-mer T cell epitope for each DENV serotype can be used to produce specific antibody against dengue virus and a chimeric common tetravalent vaccine candidate to yield a comparative vaccine to cover any of the four dengue virus serotype. This vaccine is expected to be highly immunogenic against dengue fever.

## Introduction

Dengue fever (DF), also known as break bone fever, is an infectious tropical disease caused by closely related four dengue virus serotypes (DENV1-4). Symptoms of DF include fever, headache, muscle and joint pains, and a characteristic skin rash^[1]^. In a small proportion of cases, the disease develops into life-threatening dengue hemorrhagic fever (DHF) resulting in bleeding, low levels of blood platelets and plasma leakage, or into dengue shock syndrome (DSS), where dangerously low blood pressure occurs.

Currently there are no specific treatments for DF^[2]^ and only management of the fever is given such as nursing care and maintaining fluid balance and electrolytes^[3]^. Treatment of acute dengue uses either oral or intravenous rehydration for mild or moderate disease, and intravenous fluids and blood transfusion in more severe cases. For the prevention of dengue, there is no commercially available vaccine; prevention is sought by reducing the habitat of mosquitoes and limiting exposure to bites. The *E*-protein on the viral membrane of dengue virus binds to receptors on the host cell membrane^[4]^. It is also a major antigen inducing host protective immunity with production of a neutralizing antibody^[5^–^6]^. During a viral infection, the adsorption of viral particles is initiated by binding of *E*-protein to receptor molecules present on the host cell membrane. Subsequently, the adsorbed viruses are taken into the cell by endocytosis. Development of a vaccine against dengue is difficult since there are four closely related, but antigenically distinct serotypes of the virus that can cause disease^[7^–^8]^ Infection by one serotype that induces immunity does not ensure protection of the patient from infection by the other three serotypes^[9]^ . Therefore, if vaccines for only one or two serotypes are developed, other serotypes would increase the risk of a more serious illness^[10]^. A possible strategy in the treatment of dengue is to use chimeric tetravalent vaccines that shows high neutralizing antibody titers against all dengue serotypes^[11^–^12]^. Studies on the development of tetravalent vaccines are ongoing in various parts of the world^[13]^.

Currently, effective preventive medicines and defensive immune mechanisms are not available. The vaccine produced in the conventional broad based method does not function properly. Only the vaccine, based on the specific part of the antigen alone will function properly, for which reverse vaccinology, a new method for identification of vaccine candidate, has been proposed. Hence, in the present work, reverse vaccinology has been applied to develop a potential vaccine candidate from the whole antigen. In this technique, instead of whole antigen, a part of the antigen known as T cell epitope, which is recognized by both B cell and T cell mediated immunity, was predicted.

## Materials and methods

### Prediction of antigenic B cell epitopes

The *E*-proteins of DENV 1-4 serotypes of the *Flavivirus* were selected for the current study and a novel approach of epitope designing was adopted where an epitope should produce both the B cell and T cell mediated immunity. The complete amino acid sequence of each protein was retrieved from Swiss-Prot protein database^[14]^ and analyzed using VaxiJen v2.0 antigen prediction server^[15]^ Proteins having VaxiJen score>0.4 were selected. Each selected full length amino acid sequence was then subjected to transmembrane topology analysis using TMHMM v0.2 (www.cbs.dtu.dk) prediction server^[16]^ in order to identify exomembrane amino acid sequences of each protein. For prediction of B cell epitopes, each full length protein sequence was subjected to BCPreds analysis^[17]^ and all predicted B cell epitopes (20-mers) having a BCPreds cutoff score>0.8 were selected. Selected B cell epitopes were then subsequently checked for membrane topology by comparing with TMHMM results for exomembrane amino acid sequences. Surface exposed B cell epitope sequences having the cutoff value for BCPreds (>0.8) were selected and further analyzed using VaxiJen threshold= 0.4, ACC output) to check the antigenicity. Finally, 2-3 epitopes with top VaxiJen scores were selected for use in prediction of T cell epitopes^[18]^.

### Prediction of T cell epitopes from B cell epitopes

T cell epitopes were predicted from the selected B cell epitopes. Both the sequence based and structure based QSAR simulation approaches were taken into account to predict T cell epitopes and two screening steps were adopted. In the first screening, the selection criteria were: i) the sequence should bind to both the MHC class-I and class-II molecules and the minimum number of total interacting MHC molecules should be>15, ii) the sequence must interact with HLADRB1* 0101 of MHC class-II, and iii) should be antigenic based on VaxiJen score. Propred-1 (47 MHC Class-I alleles)^[19]^ and Propred (51 MHC Class-II alleles)^[20]^ servers that utilize amino acid position coefficients inferred from literature employing linear prediction model^[21]^ were used to identify common epitopes that bind to both the MHC class molecules as well as to count five total numbers of interacting MHC alleles. For the QSAR simulation approach, the half maximal (50%) inhibitory concentration (IC_50_) and antigenicity of common epitopes predicted by Propred-1 and Propred was calculated using MHCPred v.2^[22]^ server (selecting DRB1*0101) and VaxiJen, respectively. Epitopes with the highest antigenicity and those bind more than 15 MHC molecules comprising of both the MHC class I and II alleles and less than 100 Nm IC_50_ scores for DRB1*0101 were selected. The second screening was based on structure and QSAR simulation methods using T-Epitope Designer^[23^-^24]^ and MHCPred, respectively. The final list of epitopes was made with non-overlapping peptide sequences that pass these above mentioned criteria and VaxiJen and IC_50_ scores. Selected epitopes were further analyzed for fold level topology.

### Epitope analysis

The 3D folding and clusters of epitopes in folded protein were analyzed to confirm the exomembrane topology of these epitopes using the Pepitope server^[25]^. All identified epitopes from the same protein to analyze the linear alignment of epitopes on the corresponding protein and to determine the epitope clusters and exomembrane position of epitopes in the folded proteins.

## Results

### Potentiality of ***E***-proteins as antigens

*E*-proteins of dengue virus having antigenic score>0.4 were predicted as antigenic. From the results obtained, it was found that all of the four *E*-proteins were antigenic and the VaxiJen scores ranged from 0.6723 to 0.7459 (***Table 1***). Out of four *E*-proteins, DENV-1 showed the highest VaxiJen score (0.7459). The basic criterion of a good antigen is that it must be exposed outside the membrane. The results of the transmembrane topology analyses revealed that all of the four *E*-proteins were fully exposed outside the membrane, and thereby qualified as antigenic. Thus, the four *E*-proteins showed antigenic property by having VaxiJen score greater than the threshold value (0.4) and the exomembrane topology (***Table 1***). T cell epitopes predicted from *E*-proteins through B cell epitopes elicit and stimulate anti-DENV response and are useful in vaccination and immunization as well as for treatment. Therefore, T cell epitopes were predicted from *E*-proteins of DENV-1 to DENV-4.

**Tab.1:** Antigenicity of*** E***-proteins of DENV serotypes.

No.	Proteins	VaxiJen score	Topology	Antigenicity
1	*E*-protein of DENV-1	0.7459	Exomembrane	Antigen
2	*E*-protein of DENV-2	0.6749	Exomembrane	Antigen
3	*E*-protein of DENV-3	0.7395	Exomembrane	Antigen
4	*E*-protein of DENV-4	0.6723	Exomembrane	Antigen

### B cell epitope prediction and selection

**Fig.1:**
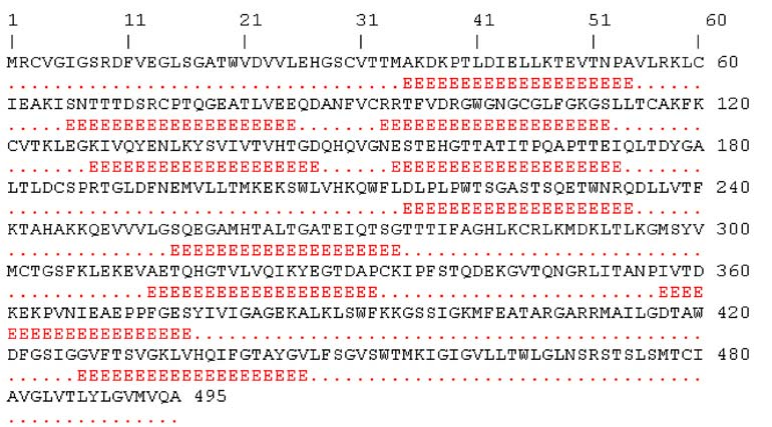
Location of B cell epitopes on whole *E*-protein antigen of DENV-1.

An antigenic peptide should produce both B cell and T cell mediated immunity for becoming a good vaccine candidate. Therefore, in the present study, to identify such epitopes, full length of all the *E*-proteins were first subjected to B cell epitope prediction using BCpreds and AAP prediction methods and all possible 20-mer B cell epitopes were predicted from each *E*-protein (***Fig. 1***). B cell epitopes having BCpred score greater than 0.8 were selected for further study. Thus, using the above prediction methods, totally 19 from DENV-1, 25 from DENV-2, 23 from DENV-3 and 26 from DENV-4 were predicted. From the predicted B cell epitopes, best B cell epitopes were selected based on BCpred score>0.8, VaxiJen score>0.4 and exomembrane topology. B cell epitopes, which fulfilled all the above three criteria, were selected as best B cell epitopes which included seven from DENV-1, eight from DENV-2, and six each from DENV-3 and DENV-4 (***Table 2***).

**Tab.2:** Prediction and selection of B-cell epitopes from full length antigens of ***E***-proteins and selection based on the scores of BCPred and VaxiJen and exomembrane topology.

B-cell epitopes	Positionof aminoacids	BCPredsscore*	VaxiJen scores **	Topology***	Antigenicity or non-antigenicity	Number of qualified B-cell epitopes
DENV-1						
BCPred method						
GNESTEHGTTATITPQAPTT	152	1	0.4837	inside	Non antigen	
**LSWFKKGSSIGKMFEATARG**	389	0.997	0.5504	outside	**Antigen**	
HAKKQEVVVLGSQEGAMHT	243	0.993	0.6644	inside	Non antigen	
**PIVTDKEKPVNIEAEPPFGE **	356	0.991	0.9472	outside	**Antigen**	
AKISNTTTDSRCPTQGEATL	63	0.990	0.8994	inside	Non antigen	
**PWTSGASTSQETWNRQDLLV **	219	0.980	0.4489	outside	**Antigen**	
RRTFVDRGWGNGCGLFGKGS	93	0.918	0.1600	inside	Non antigen	
DAPCKIPFSTQDEKGVTQNG	330	0.904	0.3398	outside	Non antigen	
**LEHGSCVTTMAKDKPTLDIE **	25	0.874	0.4534	outside	**Antigen**	
AAP method						
QNGRLITANPIVTDKEKPVN	347	1	0.4298	inside	Non antigen	
**DKPTLDIELLKTEVTNPAVL **	37	1	0.4395	outside	**Antigen**	
HQVGNESTEHGTTATITPQA	149	1	0.8033	inside	Non antigen	
AKISNTTTDSRCPTQGEATL	63	1	0.8994	inside	Non antigen	
**DLPLPWTSGASTSQETWNRQ**	215	1	0.5625	outside	**Antigen**	
SQEGAMHTALTGATEIQTSG	255	1	0.3896	outside	Non antigen	
**AETQHGTVLVQIKYEGTDAP **	313	1	0.9845	outside	**Antigen**	
KIVQYENLKYSVIVTVHTGD	128	1	0.7823	inside	Non antigen	
RRTFVDRGWGNGCGLFGKGS	93	1	0.1600	inside	Non antigen	
GVFTSVGKLVHQIFGTAYGV	427	0.996	0.2482	outside	Non antigen	7
DENV-2						
BCPred method						
**VEPGQLKLNWFKKGSSIGQM **	382	1	0.8518	outside	**Antigen**	
**PLPWLPGADTQGSNWIQKET **	217	0.994	0.5042	outside	**Antigen**	
KLTNTTTESRCPTQGEPSLN	64	0.992	1.0114	inside	Non antigen	
TVNPIVTEKDSPVNIEAEPP	353	0.987	0.9638	inside	Non antigen	
FTCKKNMKGKIVQPENLEYT	119	0.984	0.7701	inside	Non antigen	
HGTIVIRVQYEGDGSPCKIP	317	0.978	0.6115	inside	Non antigen	
AVGNDTGKHGKEIKITPQSS	150	0.968	0.9566	inside	Non antigen	
MVDRGWGNGCGLFGKGGIVT	96	0.914	0.5970	inside	Non antigen	
**LTGYGTVTMECSPRTGLDFN **	175	0.869	1.1305	outside	**Antigen**	
FELIKTEAKQPATLRKYCIE	43	0.849	0.3017	inside	Non antigen	
KNPHAKKQDVVVLGSQEGAM	241	0.813	0.7671	inside	Non antigen	
DIVLEHGSCVTTMAKNKPTL	22	0.803	0.3407	outside	Non antigen	
AAP method						
HVLGRLITVNPIVTEKDSPV	346	1	0.3941	outside	Non antigen	
**VEPGQLKLNWFKKGSSIGQM **	382	1	0.8518	outside	**Antigen**	
**GTIVIRVQYEGDGSPCKIPF **	318	1	0.5807	inside	**Antigen**	
KNKPTLDFELIKTEAKQPAT	36	1	0.3954	outside	Non antigen	
**SQEGAMHTALTGATEIQMSS **	255	1	0.6538	outside	**Antigen**	
MVDRGWGNGCGLFGKGGIVT	96	1	0.5970	inside	Non antigen	
KKNMKGKIVQPENLEYTIVI	122	1	0.6156	inside	Non antigen	
**PRTGLDFNEMVLLQMEDKAW **	187	1	1.1743	outside	**Antigen**	
AVGNDTGKHGKEIKITPQSS	150	1	0.9566	inside	Non antigen	
IEAKLTNTTTESRCPTQGEP	61	1	1.0737	inside	Non antigen	
** GISNRDFVEGVSGGSWVDIV **	5	0.997	0.8283	outside	**Antigen**	
DKLQLKGMSYSMCTGKFKIV	290	0.289	0.7080	inside	Non antigen	
FLDLPLPWLPGADTQGSNWI	213	0.097	0.3466	outside	Non antigen	8
DENV-3						
BCPred method						
TANPVVTKKEEPVNIEAEPP	351	1	1.2020	inside	Non antigen	
GIGDNALKINWYKKGSSIGK	379	0.996	0.9475	inside	Non antigen	
NAHAKKQEVVVLGSQEGAMH	240	0.993	0.7692	inside	Non antigen	
EGKITNITTDSRCPTQGEAV	62	0.963	0.9157	inside	Non antigen	
KHTYVDRGWGNGCGLFGKGS	93	0.953	0.4565	inside	Non antigen	
**VLLTWIGLNSKNTSMSFSCI **	459	0.952	1.7818	outside	**Antigen**	
**SETQHGTILIKVEYKGEDAP **	311	0.948	1.3268	outside	**Antigen**	
EITPQASTTEAILPEYGTLG	161	0.901	0.8245	inside	Non antigen	
**LEHGGCVTTMAKNKPTLDIE **	25	0.863	0.5134	outside	**Antigen**	
** EPIEGKVVQYENLKYTVIIT **	123	0.824	0.8295	outside	**Antigen**	
AAP predictions						
VTKKEEPVNIEAEPPFGESN	356	1	1.1104	inside	Non antigen	
QHQVGNETQGVTAEITPQAS	148	1	0.7463	inside	Non antigen	
**FFDLPLPWTSGATTETPTWN **	211	1	0.4613	outside	**Antigen**	
APCKIPFSTEDEQGKAHNGR	329	1	0.1066	outside	Non antigen	
QEGAMHTALTGATEIQNSGG	254	1	0.3108	outside	Non antigen	
CTNTFVLKKEVSETQHGTIL	300	1	0.1077	inside	Non antigen	
EHGGCVTTMAKNKPTLDIEL	26	1	0.5861	inside	Non antigen	
GDNALKINWYKKGSSIGKMF	381	1	0.5426	inside	Non antigen	
KTEATQLATLRKLCIEGKIT	47	1	0.8090	inside	Non antigen	
RCVGVGNRDFVEGLSGATWV	2	1	1.0117	inside	Non antigen	
YVDRGWGNGCGLFGKGSLVT	96	0.999	0.4474	inside	Non antigen	
**LEPIEGKVVQYENLKYTVII**	122	0.932	0.7026	outside	**Antigen**	
DFNEMILLTMKNKAWMVHRQ	190	0.331	0.9731	inside	Non antigen	**6**
DENV-4						
BCPred predictions						
ITTATRCPTQGEPYLKEEQD	68	0.999	0.7688	inside	Non antigen	
HGTTVIKVKYEGAGAPCKVP	317	0.983	0.8138	inside	Non antigen	
NDTSNHGVTATITPRSPSVE	153	0.973	0.7010	inside	Non antigen	
EKVVGRVISSTPFAENTNSV	345	0.930	0.2095	inside	Non antigen	
LVLEHGGCVTTMAQGKPTLD	23	0.924	0.1595	outside	Non antigen	
QIENLEYTVVVTVHNGDTHA	131	0.923	0.7298	inside	Non antigen	
**NIELEPPFGDSYIIIGVGDS **	366	0.886	0.9095	outside	**Antigen**	
**PLPWAAGADTSEVHWNYKER **	217	0.886	1.4667	outside	**Antigen**	
GMSYTMCSGKFSIDKEMAET	296	0.882	-0.1708	inside	Non antigen	
WIGTNSRNTSMAMTCIAVGG	465	0.880	1.5598	inside	Non antigen	
**ATEVDSGDGNHMFAGHLKCK **	267	0.860	0.8850	outside	**Antigen**	
**VDRGWGNGCGLFGKGGVVTC **	97	0.851	0.4260	outside	**Antigen**	
TLHWFRKGSSIGKMFESTYR	388	0.794	0.4553	inside	Non antigen	
FNEMILMKMKKKTWLVHKQW	193	0.754	0.6979	inside	Non antigen	
GKAVHQVFGSVYTTMFGGVS	433	0.752	0.2638	outside	Non antigen	
AAP predictions						
**EGAMHSALTGATEVDSGDGN **	257	1	0.4981	outside	**Antigen**	
AENTNSVTNIELEPPFGDSY	358	1	0.8180	inside	Non antigen	
VSGGAWVDLVLEHGGCVTTM	15	1	0.3032	outside	Non antigen	
**LLTSLGKAVHQVFGSVYTTM **	428	1	0.4270	outside	**Antigen**	
GKGGVVTCAKFSCSGKITGN	109	1	0.5995	inside	Non antigen	
LEYTVVVTVHNGDTHAVGND	135	1	0.2622	outside	Non antigen	
KYEGAGAPCKVPIEIRDVNK	325	1	0.8884	inside	Non antigen	
IEASISNITTATRCPTQGEP	61	1	0.7843	inside	Non antigen	
TNSRNTSMAMTCIAVGGITL	468	0.849	1.4800	inside	Non antigen	
PWAAGADTSEVHWNYKERMV	219	0.101	1.1611	outside	Non antigen	
IKGMSYTMCSGKFSIDKEMA	294	0.075	-0.1411	inside	Non antigen	6

Selected B-cell epitopes are indicated in bold.

**Fig.2:**
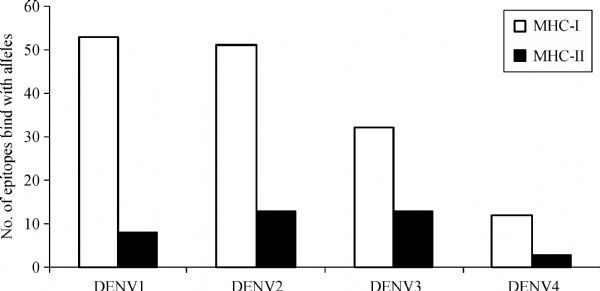
Dengue serotype epitopes bind with MHC-I and MHC-II alleles.

**Fig.3:**
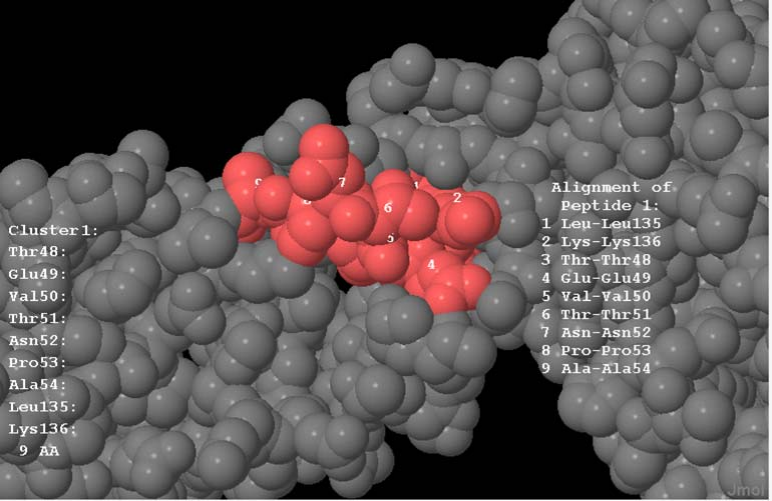
Exposed T-cell epitope (LKTEVTNPA) located on the outer surface of the ***E***-protein of DENV-1.

Each selected B-cell epitope was analyzed for the prediction of T cell epitopes within the B cell epitope sequence using the sequence based and structure based QSAR simulation approaches. The criteria used for the screening were (i) sequences those bound more than 15 MHC alleles comprising of both the MHC class I & II, using Propred, (ii) the sequences interacted with HLA DRB1*0101 with IC_50_ value less than 50 nmol/L and (iii) the epitope with the highest antigenicity. From the selected B cell epitopes of *E*-proteins, promiscuous T cell epitopes were predicted using the server Propred-1 and Propred. Fifty-three promiscuous T cell epitopes from DENV-1, 51 from DENV-2, 32 from DENV-3 and 12 from DENV-4 were predicted through the Propred-1 server for MHC class-I allele (***Fig.2***). Eight T cell epitopes from DENV-1, 13 each from DENV-2 and DENV-3, and three from DENV-4 were predicted through the Propred server for MHC class-II binding alleles (***Table 3 ***and ***Fig.2***). From the above predictions, T cell epitopes, predicted by both Propred-1 and Propred were identified. T cell epitopes, those bound with the highest number of MHC alleles comprised of both MHC class I & II, were identified. From the above study, one common T cell epitope from DENV-1, and two each from DENV-2, DENV-3 and DENV-4 were selected (***Table 4***). The IC_50_ values and antigenicity of the above selected common T cell epitopes were calculated using MHCPred, VaxiJen and exomembrane topology. Thus, at the end, totally four T cell epitopes, one each from DENV-1 (LKTEVTNPA), DENV-2 (MVLLQMEDK), DENV-3 (IGLNSKNTS) and DENV-4 (YIIIGVGDS), were selected as the best T cell epitopes. Other T cell epitopes were not selected due to a low VaxiJen score and high IC_50_ values. In DENV-3 (VVQYENLKY) and DENV-4 (LTSLGKAVH) the IC_50_ values were greater than 50 Nm and showed only intermediate affinity toward MHC alleles. On the other hand, in DENV-2 LNWFKKGSS, the VaxiJen score (0.2112) was less than the threshold value of 0.4 for which the other T cell epitopes of the DENV-2 were not qualified (***Table 5***). Selected T cell epitopes could be used as vaccine candidates which could act as an effective monovalent vaccine and be immunogenic against specific DENV serotype. The T cell epitopes were checked for their exomembrane topology and antigenicity. There is every possibility of the identified epitopes getting folded inside the tertiary structure of the corresponding protein and therefore, a fold level analysis of the proteins was carried out. The folded proteins showed that the identified T cell epitopes were situated in the clusters and located on the outer surface of the corresponding enzymes (***Fig. 3***).

**Tab.3:** **P**rediction of T-cell epitopes from selected B-cell epitopes of E-proteins.

B-cell epitopes	Epitopes binding with MHC-I alleles using propred-1	No. of epitopes	Epitopes binding with MHC-II alleles using propred	No. of epitopes
DENV-1				
BCPred method				
LSWFKKGSSIGKMFEATARG	KMFEATARG, KGSSIGKMF, KKGSSIGKM, SSIGKMFEA, SWFKKGSSI, SIGKMFEAT, IGKMFEATA		WFKKGSSIG, LSWFKKGSS, FKKGSSIGK	
PIVTDKEKPVNIEAEPPFGE	NIEAEPPFG, IVTDKEKPV, VNIEAEPPF, KEKPVNIEA, VTDKEKPVN, TDKEKPVNI		Nil	
PWTSGASTSQETWNRQDLLV	ETWNRQDLL, STSQETWNR, QETWNRQDL, TWNRQDLLV, WTSGASTSQ, GASTSQETW		Nil	
LEHGSCVTTMAKDKPTLDIE	GSCVTTMAK, CVTTMAKDK, TMAKDKPTL, AKDKPTLDI, EHGSCVTTM, LEHGSCVTT, TTMAKDKPT, HGSCVTTMA, MAKDKPTLD		Nil	
AAP method				
DKPTLDIELLKTEVTNPAVL	TLDIELLKT, PTLDIELLK, KPTLDIELL, DIELLKTEV,		IELLKTEVT, **LKTEVTNPA**	
DLPLPWTSGASTSQETWNRQ	DKPTLDIEL, **LKTEVTNPA,** TEVTNPAVL, KTEVTNPAV		**LKTEVTNPA**	
AETQHGTVLQIKYEGTDAP	STSQETWNR, LPLPWTSGA, GASTSQETW, SGASTSQET	53	Nil	5
DENV-2				
BCPred method				
VEPGQLKLNWFKKGSSIGQM	GTVLVQIKY, LVQIKYEGT, AETOQHGTVL, QHGTVLVQI, ETQHGTVLV		IKYEGTDAP, VQIKYEGTD, VLVQIKYEG	
PLPWLPGADTQGSNWIQKET	GOLKLNWFKK, **LNWFKKGSS, **EPGQLKLNW, VEPGQLKLN, KKGSSIGQM, NWFKKGSSI		FKKGSSIGQ, **LNWFKKGSS, **WFKKGSSIG	
LTGYGTVTMECSPRTGLDFN	TQGSNWIQK, **LPGADTQGS, **ADTQGSNWI, LPWLPGADT, LPGADTQGS, GSNWIQKET		**LPGADTQGS**	
AAP method				
VEPGQLKLNWFKKGSSIGQM	TVTMECSPR, GYGTVTMEC, CSPRTGLDF, MECSPRTGL, **LTGYGTVTM, **SPRTGLDFN, YGTVTMECS		**LGTYGTVTMFKKGSSIGQ, **LNWFKKGSS	
GTIVIRVQYEGDGSPCKIPF	**WFKKGSSIC**, GQLKLNWFK, EPGQLKLNW		**WFKKGSSIC,**	
SQEGAMHTALTGATEIQMSS	KKGSSIGQM, VEPGQLKLN, NWFKKGSSI,** FKKGSSIGQ, **GTIVIRVQY, DGSPCKIPF			
PRTGLDFNEMVLLQMEDKAW	QYEGDGSPC, GTIVIRVQY, **YEGDGSPCK**, GTIVIRVQY, EGDGSPCKI		**YEGDGSPCK, **IVIRVQYEG	
GISNRDFVEGVSGGSWVDIV	AMHTALTGA, SQEGAMHTA, LTGATEIQM, QEGAMHTAL, MHTALTGAT, TALTGALTEI	51	Nil	13
DENV-3				
BCPred method				
VLLTWIGLNSKNTSMSFSCI	**MVLLQMEDK**, TGLDFNEMV, GLDFNEMVL, RTGLDFNEM, LDFNEMVLL, **FNEMVLLQM, **LLQMEDKAW		**MVLLQMEDK, FNEMVLLQM**	
SETQHGTILIKVEYKGEDAP	**FVEGVSGGS,** SGGSWVDIV, NRDFVEGVS, VSGGSWVDI, VEGVSGGSW		**FVEGVSGGS**	
LEHGGCVTTMAKNKPTLDIE	GLNSKNTSM,** IGLNSKNTS, WIGLNSKNT, **LTWIGLNSK, SKNTSMSFS, VLLTWIGLN, NSKNTSMSF, NTSMSFSCI		**WIGLNSKNT**, IGLNSKNTS,LNSKNTSMS, LLTWIGLNS	
EPIEGKVVQYENLKYTVIIT	**ILIKVEVKG**, TQHGTILIK, TILIKVEYK, GTILIKVEY, QHGTILIKV		IKVEYKGED, **ILEKVEYKG**	
AAA predictions				
FFDLPLPWTSGATTETPTWN	TMAKNKPTL, CVTTMAKNK		Nil	
LEPIEGKVVQYENLKYTVII	AKNKPTLDI, EHGGCVTTM, LEHGGCVTT, **VVQYENLKY,** GKVVQYENL	32	**YEMLKYFVI, VVQYENLKY, IEGKVVQYE**	9
DENV-4				
BCPred predictions				
NIELEPPFGDSYIIIGVGDS	VQYENLKYT**, **KVVQYENLK, QYENLKYTV, ENLKYTVII, GKVVQYENL		Nil	
PLPWAAGADTSEVHWNYKER	**YENLKYTVI, LEGKVVQYE**		**YENLKYTVI**, LEPIEGKVV	
ATEVDSGDGNHMFAGHLKCK	LPLPWTSGA, FDIPLPWTS		Nil	
VDRGWGCGLFGKGGVVTTC	**VVQYENLKY, IEGKVVQYE**		**VVQYENLKY, IEGKVVQYE**	
AAP predictions				
EGAMHSALTGATEVDSGDGN	VQYENLKYT, **YIIIGYGDS**		**YIIIGYGDS**	
LLTSLGKAVHQVFGSVYTTM	TSEVHWNYK, EVHWNYKER	12	Nil	
	SGDGNHMFA, EVDSGDGNH, GNHMFAGHL		Nil	
	**FGKGGVVTC**		**FGKGGVVTC**	
	ATEVDSDSGDG, AMHSALTGA,		Nil	
	TEVDSGDGN, SALTGATEV, **LTSLGKAVH**		**LTSLGKAVH**	13

Common T-cell epitopes predicted by both MHC-I and MHC-II alleles shown in bold.

**Tab.4:** Selection of qualified T-cell epitopes from ***E***-proteins showing highest binding with MHC I and II alleles

Selected B-cell epitopes	Common T-cell Epitopes for both MHC I and II	No. of alleles bound with MHC-I alleles	No. of alleles bound with MHC-II alleles	Total No. of alleles (MHC-I+II)	Qualified T-cell epitopes with highest binding (MHC I+II) Alleles
DENV-1					(1)
BCPred method	Nil	-	-	-	-
AAP method					
DKPTLDIELLKTEVTNPAVL	LKTEVTNPA	1	7	8	LKTEVTNPA
DENV-2					(2)
BCPred method					
VEPGQLKLNWFKKGSSIGQM	LNWFKKGSS	1	15	16	LNWFKKGSS
PLPWLPGADTQGSNWIQKET	LPGADTQGS	4	3	7	Nil
LTGYGTVTMECSPRTGLDFN	LTGYGTVTM	5	3	8	Nil
AAP method					
PRTGLDFNEMVLLQMEDKAW	MVLLQMEDK	5	10	15	MVLLQMEDK
PRTGLDFNEMVLLQMEDKAW	FNEMVLLQM	1	7	8	Nil
GISNRDFVEGVSGGSWVDIV	FVEGVSGGS	5	2	7	Nil
DENV-3					(2)
BCPred method					
VLLTWIGLNSKNTSMSFSCI	IGLNSKNTS	1	27	28	IGLNSKNTS
	WIGLNSKNT	4	5	9	Nil
SETQHGTILIKVEYKGEDAP	ILIKVEYKG	1	1	2	Nil
EPIEGKVVQYENLKYTVIIT	VVQYENLKY	13	18	31	VVQYENLKY
	IEGKVVQYE	1	4	5	Nil
	YENLKYTVI	2	1	3	Nil
AAP predictions					
LEPIEGKVVQYENLKYTVII	IEGKVVQYE	1	4	5	Nil
DENV-4					(2)
BCPred predictions					
NIELEPPFGDSYIIIGVGDS	YIIIGVGDS	2	37	39	YIIIGVGDS
VDRGWGNGCGLFGKGGVVTC	FGKGGVVTC	4	2	6	Nil
AAP predictions					
LLTSLGKAVHQVFGSVYTTM	LTSLGKAVH	8	14	22	LTSLGKAVH

**Tab.5:** Selection of T-cell epitopes from qualified T-cell epitopes of *E*-proteins based on VaxiJen score, IC_50_ value and exomembrane topology

Qualified T-cell epitopes	VaxiJen score of T-cell epitopes	MHCpred score (Ic_50_ value) of selected T-cell epitopes	Topology of T-cell epitope	Selected T-cell epitopes
DENV-1				
LKTEVTNPA	0.4019	56.23	Exomembrane	LKTEVTNPA
DENV-2				
LNWFKKGSS	0.2112	44.67	-	Not qualified
MVLLQMEDK	1.6436	210.38	Exomembrane	MVLLQMEDK
DENV-3				
IGLNSKNTS	2.2818	62.23	Exomembrane	IGLNSKNTS
VVQYENLKY	1.0916	327.34	-	Not qualified
DENV-4				
YIIIGVGDS	0.9665	20.99	Exomembrane	YIIIGVGDS
LTSLGKAVH	0.6897	144.88	-	Not qualified

Since monovalent 9-mer epitope elicits immunity against single DENV serotype, a chimeric tetravalent vaccine, formed by the conjugation of the above four T cell epitopes from four DENV serotypes, is preferred over monovalent T cell epitope. This tetravalent vaccine is expected to prevent DF caused by any of the four serotypes of dengue viruses. This tetravalent vaccine was tested for its antigenic nature by using VaxiJen and TMHMM and found to be highly antigenic by having the antigenic score 1.1927 and the entire length of tetravalent vaccine was fully exomembrane in topology.

### Confirmation of vaccine candidates for vaccine production

To find out the ability of the vaccine candidates for their immunogenic response to produce antibodies, the subunit vaccine candidates of DENV-1 (LKTEVTNPA), DENV-2 (MVLLQMEDK), DENV-3 (IGLNSKNTS) and DENV-4 (YIIIGVGDS) were submitted to the Peptide Station at Sweden for verification through online Peptide Design Tool. The suitability of vaccine candidate to act as subunit vaccine was studied based on water solubility, peptide length, weight, conjugation ability and modifications of the termini. Each vaccine candidate was conjugated with a carrier molecule containing single cysteine which acted as a coupling site. Furthermore, the carrier molecule is conjugated with the peptide sequence (vaccine candidate) for the production of peptide vaccine. The four vaccine candidates, DENV-1(LKTEVTNPA, DENV-2 (MVLLQMEDK), DENV-3 (IGLNSKNTS) and DENV-4 (YIIIGVGDS), according to Peptide Station at Sweden, were identified as effective immunogenic vaccine candidates for antibody production. Such antibodies could be used to treat the patient affected by specifically known serotypes of Dengue virus. A similar study was also carried out using the tetravalent vaccine LKTEVTNPAMVLLQMEDKIGLNSKNTSYIIIGVGDS to act as a common vaccine candidate against four serotypes of dengue virus to combat DF, DHF and DSS. The tetravalent vaccine candidate, LKTEVTNPAMVLLQMEDKIGLNSKNTSYIIIGVGDS was also submitted to the Peptide Station at Sweden for verification through online Peptide Design Tool. They identified this tetravalent vaccine candidate as an effective immunogenic vaccine candidate for antibody production. This tetravalent vaccine could be used for vaccination against all the four serotypes of DENV which will prevent any DF infection.

## Discussion

### Whole antigen

Increased efforts are needed in the development of an effective vaccine against DENV. There are four antigenically related serotypes (dengue-1, -2, -3, and -4) that are known to cause DF^[25]^. During viral infection, the adsorption of viral particles is initiated by binding of *E*-protein to receptor molecules present on the host cell membrane. Subsequently, the adsorbed viruses are taken into the cell by endocytosis. *E*-protein plays important roles during the life cycle of the dengue virus. *E*-protein is a major antigen against host protective immunity, which induces neutralizing antibody^[26^–^27]^. The envelope (E) glycoprotein having 495 amino acids has been reported to play an important role in the dengue virus attachment to the host cell receptors and entry into the target cells^[28]^. Increased efforts are needed in the development of effective monoclonal antibodies for the treatment and vaccine for the prevention of disease through immunization. Therefore, *E*-protein is the most obvious attractive target for the production of therapeutic monoclonal antibodies and vaccine for immunization. Hence, in the present study, among the three structural proteins, C, M and E, envelop protein has been selected for *in silico* development of vaccine candidate.

### Epitopes

For effective antigenicity, the antigen should not be a lengthy one. An ideal antigen should be 8 to 20 amino acid residues in length. Such short peptide segment is described as an epitope. An epitope with 20 amino acid residues is termed as B cell epitope and an epitope with nine amino acid residues as T cell epitope-driven approaches for vaccine design are comparatively more useful and safe as they have no lethal effect. An epitope is the part of an antigen that is recognized by the immune system, such as antibodies, B cells and T cells. Most antigens have the potential to induce several distinct antibodies, each of which is specific to a particular epitope in that antigen. The prediction systems in humoral epitope discovery are still in their infancy, but in cellular immunology, MHC binding predictions are now very strong and cover most of the known HLA specificities. These systems work well for epitope discovery^[29]^.

### B cell epitopes

Antibodies bind to antigens at specific sites corresponding to the antigenic determinants or B cell epitopes. B cell epitope prediction is a highly challenging field. Computational tools for reliably predicting linear B cell epitopes in protein sequences are highly desirable, because experimental determination of epitopes is expensive in terms of cost, time, and effort involved. Several computational methods for B cell epitope prediction have been developed in recent years^[30]^. The BCpred server allows users to choose the method for predicting B cell epitopes among several developed prediction methods. The current implementation of BCpred allows the user to select two prediction methods such as implementation of AAP and BCpred^[31]^. 

In the present study, all the *E*-proteins of dengue virus serotypes were antigenic in nature. In the whole antigens of *E*-protein sequences the promiscuous T cell epitopes are present. Therefore, the T cell epitope prediction through B cell epitopes was carried out from the whole antigens. The aim of the present study was to find out highly antigenic and immunogenic T cell epitope vaccine candidate to elicit or induce B cell and T cell mediated immunity for which B cell epitope prediction is necessary. Therefore, initially, B cell epitopes, which can induce only B cell mediated immunity, are predicted. Among B cell epitopes, highly antigenic B cell epitopes were selected to predict T cell epitopes. As a result, best B cell epitopes of *E*-proteins of DENV-1, DENV-2, DENV-3 and DENV-4, were selected. Earlier studies have reported either B cell or T cell mediated epitope designing for a given pathogen^[32^–^34]^. Even though, the identification and characterization of B cell epitopes in antigens are the key steps in epitope driven vaccine design and antibody production derived T cell epitope directly from the whole antigen sequences without considering B cell epitope prediction^[35]^. On the other hand, the proposed basic epitope prediction strategy to get the minimum number of antigenic epitopes that are capable of producing both the B cell and T cell mediated immunity^[36]^. An epitope that can produce both B cell and T cell (MHC I and MHC II) mediated immunity is very useful in developing a highly immunogenic peptide-based vaccine candidate. To design and develop such a peptide vaccine against the pathogen, computational approach has been adopted based on sequence, structure, QSAR, and simulation methods along with fold level analysis to predict potential antigenic B cell epitope derived T cell epitopes^[37]^. BCpreds and AAP methods and the T cell epitopes were predicted only from the best B cell epitopes as done by Barh *et al.*^[38]^.

### T cell epitopes

The role of the immune system is to protect the host against diseases by identifying and killing pathogens through the immune system. T cell epitopes typically are short amino acid sequences, nine amino acids in length and are sufficient to increase, stimulate, promote, enhance or induce anti-dengue virus T cell response. A new strategy for developing prophylactic and therapeutic application of pathogen specific immunity is provided by the epitope based vaccines. It is a critical requirement for the identification and selection T cell epitopes that act as vaccine target. Several epitopes were predicted and identified in the structural proteins. 

A new approach of vaccine design is now emerging and following essential discoveries in immunology and the development of bioinformatics tools for T cell epitope prediction from the protein sequences. Many web based tools are publically available for predicting T cell epitopes from the protein sequence. The present study was conducted to predict and identify the promiscuous epitope that binds to large number of HLA molecules computationally. The need for dengue vaccine that elicits sustained levels of antibodies and provide long lasting protection in vaccinated people without any risk to their health either by producing adverse effects or by inducing low levels of antibodies^[39^–^43]^ while studying antigenic B cell epitopes in *N. gonorrhoeae,* predicted and selected 20-mer B cell epitopes from which they predicted T cell epitopes. Such T cell epitopes are ideal antigenic peptides which are able to produce both B cell and T cell mediated immunity. T-lymphocytes play a major role in recognizing and subsequent elimination of diseases. Induction of epitope-specific T cell responses can help in the clearance of diseases for which no conventional vaccines exist. However, (i) the lack of simple methods to identify relevant T cell epitopes, (ii) the high mutation rate of many pathogens, and (iii) HLA polymorphism have made the development of efficient T cell epitope-based vaccines difficult to achieve^[29]^. 

Due to the availability of several host and pathogen genomes and numerous tools for *in silico* prediction of effective B cell and T cell epitopes, recent trend of vaccine designing has been shifted to peptide or epitope based subunit vaccines that are more specific, safe, and easy to produce. To design and develop such a peptide vaccine against the pathogen, a novel computational approach has been adopted to predict potential antigenic B cell epitope derived T cell epitopes^[40]^. In identifying the ideal T cell epitopes, two graphical web tool servers play a major role in determining the promiscuous T cell epitopes. They are Propred-1 (47 MHC Class-I alleles) and Propred (51 MHC Class-II alleles) which were used to identify common T cell epitopes that could interact with both the MHC classes with the highest number of alleles and thus could produce better antigenic response^[34^,^37^,^43^–^45]^. In this process, only 9-mer peptides were predicted^[46]^. Recently, in many studies, B cell epitope has been analyzed for the prediction of T cell epitopes within the B cell epitope sequence that could interact with both the MHC classes with the highest number of alleles and thus could produce better antigenic response^[46]^.

### Vaccine

A vaccine is a biological preparation that improves immunity to a particular disease, which typically contains an agent that resembles a disease-causing microorganism, and is often made from weakened or killed form of the microbe. Vaccination studies in dengue model have provided insights into how immunoprophylaxis confers protection. Specific antibody conferred immunity by increasing viral load clearance and by inhibiting the viral attachment to the host cell, a crucial early step in the pathogenesis of this disease^[47]^. However, they used whole antigen as a vaccine candidate instead of peptide based subunit vaccine. Epitope driven vaccine design is comparatively more useful and safe than the whole protein vaccines as they have no lethal effect^[48]^. Thus, the peptide vaccine candidate selected in this study would be more effective in producing immunogenic response over the whole antigen against dengue. The National Institute of Allergy and Infectious Diseases (NIAID) of the USA has recognized different types of vaccines such as live, attenuated, inactivated, subunit, toxoid, conjugate, DNA based and recombinant vector vaccines. Among them, sub unit vaccines were considered to be more effective because they included only the 9-mer antigens, the very specific parts of the antigen that avoid unwanted side effects caused by the whole antigen^[49]^.

Hence, in the present study, prediction of monovalent vaccine, in the form of T cell epitope was done in order to induce specific antibody in the immune system of the host organism. Vaccination against viral pathogens has commonly been used to prevent and treat infectious diseases most effectively. A number of proteins such as E and NS antigens are available as promising vaccine targets. These antigens have frequently been used in the development of effective vaccines which will eventually be the predominant alternative approach for treatment and prevention of dengue (http://www.uniprot.org/).

### Monovalent vaccine

Development of vaccine for dengue is a complex process, since dengue is caused by four dengue serotypes, DENV-1, DENV-2, DENV-3 and DENV-4. In this case, it is difficult to predict which type of dengue is prevalent in the particular geographical region for conducting vaccination program to prevent dengue. At present, tetravalent vaccine formulation is in progress for which monovalent vaccine identification for all the four dengue serotypes are essential^[50]^. Therefore, in the present study, the monovalent vaccine candidate, T cell epitope for each dengue serotype, was predicted from whole antigen through B cell epitope prediction. From the selected B cell epitopes of *E*-protein, promiscuous T cell epitopes were predicted using the server Propred-1 and Propred for MHC class I and II binding. From the predicted T cell epitopes, common promiscuous T cell epitopes were identified from each of the *E*-proteins. Thus, at the end, totally four T cell epitopes, one each from DENV-1 (LKTEVTNPA), DENV-2 (MVLLQMEDK), DENV-3 (IGLNSKNTS) and DENV-4 (YIIIGVGDS) were selected as the best T cell epitopes. These T cell epitopes could be used as monovalent vaccine candidates which could act as an effective vaccine and be immunogenic against DENV serotypes.

### Tetravalent vaccine

A successful vaccine against dengue virus must have two characteristic features. (i) It must induce immunity against all four dengue virus serotypes simultaneously. (ii) It must induce long lasting antibody responses. A tetravalent dengue vaccine is one in which four monovalent vaccine candidates are conjugated. The vaccine elicits neutralizing antibodies to all four dengue serotypes^[51]^. Such a tetravalent vaccine is expected to satisfy the above two characteristics. In the recent past, the tetravalent construction was done differently by different people. A tetravalent dengue DNA vaccine, in which four monovalent vaccines were mixed, was formulated in a non-lipid adjuvant and tested in rhesus monkeys. The vaccine elicited neutralizing antibodies to all four dengue serotypes^[52]^. As previously stated, a dengue vaccine must produce protective immune responses to all four dengue serotypes. This obviously increases the complexity of the vaccine and the costs associated with production. The novel gene shuffling technology was used to produce individual DNA vaccine constructs that express chimeric E antigen containing epitopes from all four dengue serotypes. Many such shuffled tetravalent vaccine constructs were immunogenic in mice and produced neutralizing antibodies against all four serotypes^[53]^. These vaccine constructs also produced neutralizing antibodies to all four serotypes in vaccinated rhesus monkeys^[54]^.

To express the complete *E*-protein in its natural conformation, Wilsona *et al.*^[55]^ developed two bivalent vaccine constructs, one expressing prM and E genes of DV-1 and DV-2 and the other those of DV-3 and DV-4. These bivalent vaccines construct elicited bivalent neutralizing antibodies in vaccinated mice. A tetravalent DV vaccine formulated by mixing the two bivalent constructs was recently tested in a rhesus monkey model. Vaccinated animals not only produced high titer neutralizing antibodies against all four DV serotypes, but also the immunization completely protected the animals from viremia when challenged with any of the four DV serotypes. Both short-term (one month after vaccination) and long-term (six months after vaccination) protection were demonstrated. 

Candidate vaccines have been tested as monovalent (single virus), bivalent (two viruses), trivalent (three viruses) and tetravalent (all four serotype viruses) vaccines in Thai volunteers. They were found to be safe and immunogenic in both adults and children^[56]^. Four serotypes of monovalent live attenuated dengue virus vaccine candidates were tested for immunogenicity, which were then combined into a tetravalent formulation and given to ten volunteers. A high-titer neutralizing antibody responses were observed in volunteers who received tetravalent vaccines^[57]^. In the present investigation, monovalent 9-mer epitope elicits immunity against single DENV serotype. A tetravalent vaccine, formed by a combination of four T cell epitopes from four DENV serotypes, was formulated for analysis through bioinformatics. This tetravalent vaccine is expected to prevent all the four dengue serotypes. In this study, all the four T cell epitopes, selected to act as monovalent vaccines, were conjugated to act as a compound tetravalent vaccine. The formulated tetravalent vaccine construct was sent to Peptide station at Sweden to further investigate its ability to elicit immunogenicity and antibody production against all the four dengue virus serotypes. The tetravalent vaccine was shown to be able to produce antibodies against all the four dengue serotypes. The T cell epitopes selected to act as vaccine candidates can be incorporated into a pharmaceutical composition in a pharmaceutically acceptable carrier like water, ethanol, synthetic oils, adjuvants like oil, co-solvents such as isopropyl alcohol, glycerol etc, and such pharmaceutical compositions are useful for administering the vaccine to the patient.

## Conclusion

From the above discussion, it is concluded that 9-mer T cell epitopes are ideal peptide vaccine candidates to elicit immunity. The monovalent vaccines are useful for the production of specific antibody to treat patients affected by specific serotypes of dengue virus. On the other hand, tetravalent chimeric vaccine could be used for immunization to prevent population against DF affected by any of the four DENV serotypes.
